# *IGH::NSD2* Fusion Gene Transcript as Measurable Residual Disease Marker in Multiple Myeloma

**DOI:** 10.3390/cancers16020283

**Published:** 2024-01-09

**Authors:** András Bors, András Kozma, Ágnes Tomán, Zoltán Őrfi, Nóra Kondor, Szabolcs Tasnády, István Vályi-Nagy, Péter Reményi, Gábor Mikala, Hajnalka Andrikovics

**Affiliations:** 1 Laboratory of Molecular Genetics, Central Hospital of Southern Pest-National Institute of Hematology and Infectious Disease, H-1097 Budapest, Hungary; kozma.andras@dpckorhaz.hu (A.K.); agnes.toman89@gmail.com (Á.T.); orfi.zoltan@dpckorhaz.hu (Z.Ő.); andrikovics.hajnalka@dpckorhaz.hu (H.A.); 2 Department of Hematology and Stem Cell Transplantation, Central Hospital of Southern Pest-National Institute of Hematology and Infectious Disease, H-1097 Budapest, Hungary; tasnady.szabolcs@dpckorhaz.hu (S.T.); drvnistvan@gmail.com (I.V.-N.); premenyi@dpckorhaz.hu (P.R.); gmikala@dpckorhaz.hu (G.M.)

**Keywords:** multiple myeloma, measurable residual disease, real-time PCR, digital PCR, t(4;14), *IGH::NSD2*, *MMSET*, MB4 breakpoints, overall survival

## Abstract

**Simple Summary:**

Multiple myeloma is the second most common malignant hematologic cancer. In approximately 15% of cases, a chromosomal abnormality, t(4;14), leads to uncontrolled production of a protein called NSD2. Different-sized fragments may be missing from the initial segment of this protein, resulting in the creation of three types of breakpoints in the gene encoding the protein. Examining the level of complex (fusion) mRNAs responsible for protein formation might be crucial in the treatment of patients, as lower levels could be associated with a more favorable survival outcome. We have developed a quantitative polymerase chain reaction system capable of detecting measurable residual disease with a sensitivity of up to 1:100,000. Our study also demonstrated that the survival of patients with different breakpoint mRNAs may vary. Among them, those with intermediate-length mRNAs have the worst outcomes.

**Abstract:**

Multiple myeloma (MM) is the second most common hematological malignancy. Approximately 15% of MM patients are affected by the t(4;14) translocation resulting in the *IGH::NSD2* fusion transcript. Breakage occurs in three major breakpoint regions within the *NSD2* gene (MB4-1, MB4-2, and MB4-3), where MB4-1 leads to the production of full-length protein, while truncated proteins are expressed in the other two cases. Measurable residual disease (MRD) has been conclusively established as a crucial prognostic factor in MM. The *IGH::NSD2* fusion transcript can serve as a sensitive MRD marker. Using bone marrow (BM) and peripheral blood (PB) samples from 111 patients, we developed a highly sensitive quantitative real-time PCR (qPCR) and digital PCR (dPCR) system capable of detecting fusion mRNAs with a sensitivity of up to 1:100,000. PB samples exhibited sensitivity three orders of magnitude lower compared to BM samples. Patients with an MB4-2 breakpoint demonstrated significantly reduced overall survival (*p* = 0.003). Our novel method offers a simple and sensitive means for detecting MRD in a substantial proportion of MM patients. Monitoring may be carried out even from PB samples. The literature lacks consensus regarding survival outcomes among patients with different *NSD2* breakpoints. Our data align with previous findings indicating that patients with the MB4-2 breakpoint type tend to exhibit unfavorable overall survival.

## 1. Introduction

Multiple myeloma (MM) is the second most common incurable hematologic malignancy, originating from terminally differentiated B-cells primarily residing in the bone marrow. During the progression of the disease, clonal cells may acquire the capacity to proliferate at sites outside the bone marrow, possibly leading to extramedullary myeloma and plasma cell leukemia. The genome of MM is complex and heterogeneous; translocations between the immunoglobulin heavy chain (*IGH*) locus and recurrent oncogenes (approximately 40% of all cases), as well as hyperdiploidy (about 60% of patients), are considered early driver factors. The t(4;14) translocation, generating the *IGH::NSD2* gene fusion, is one of the most commonly identified translocations at the time of diagnosis, affecting approximately 15% of patients. This translocation universally leads to high expression of histone methyltransferase nuclear SET domain-containing 2 (*NSD2*), also known as *MMSET*, and it plays a pivotal role in the evolution of MM, tumor progression, and genomic instability [1,2,3].

While proteasome inhibitor-based regimens offer significant benefits to the majority of patients with t(4;14), low complete response (CR) rates and resistance to chemotherapies remain significant challenges. Measurable residual disease (MRD) status is an independent variable for prognostic impact and disease outcome [4,5,6]. In general, MRD can be studied in bone marrow samples using multiparameter flow cytometry immunophenotyping and molecular approaches. Currently available molecular techniques include allele-specific oligonucleotide-dependent polymerase chain reaction (ASO-dependent PCR) detected by quantitative (qPCR) or potentially digital PCR (dPCR) and next-generation sequencing (NGS), which offers high sensitivity (down to 10^5^, which means the persistence of one tumor cell in ≥10^5^ normal cells) and has been fully standardized by the EuroMRD group [7,8]. These molecular tests are based on the detection of rearranged *IGH* alleles that are unique to the malignant B-cell clone in each patient. Novel methods using liquid biopsies with circulating tumor cells and cell-free DNA show promise as future non-invasive biomarkers for detecting the mutational landscape of MM and monitoring response to therapy [9,10].

In addition to the aforementioned *IGH* gene rearrangements as molecular targets, it is also possible to monitor the presence of *IGH::NSD2* gene fusion transcripts. This would offer the potential to detect MRD in nearly 15% of MM patients without the need for patient-specific ASO-PCR tests or NGS measurements. The investigation of the *IGH::NSD2* fusion transcript is complicated by the fact that three breakpoints within the 5’ coding region of the *NSD2* locus occur (referred to as MB4-1, MB4-2, and MB4-3 in the literature). Hybrid transcripts from the MB4-1 breakpoint encode the full-length *NSD2* protein, while MB4-2 transcripts lack the first translated exon of *NSD2*. The MB4-3 transcript lacks both the first and second translated exons of *NSD2*. This may, in part, account for the prognostic heterogeneity observed in t(4;14) gammopathies [11,12].

The molecular genetic method applicable at the time of diagnosis is based on a simple qualitative PCR followed by agarose gel electrophoresis, developed in 2000 [13]. However, the same set of amplification oligos is not applicable for quantitative MRD monitoring due to the large size of PCR products. High sensitivity was achieved with the use of nested PCR primers. A single qPCR method for *IGH::NSD2* published in 2007 stated no evidence about the sensitivity [14]. No standardized consensus qPCR method for the detection of *IGH::NSD2* mRNA is available, which is similar to the recommended qPCRs in chronic and acute leukemias published in the frame of the Europe Against Cancer program [15].

Our aim was to develop a simple qPCR/dPCR system capable of detecting *IGH::NSD2* fusion mRNA with three common breakpoints of the *NSD2* gene with high sensitivity. We intended to test our newly developed method on numerous samples taken at different time points from a large cohort of t(4:14)-positive myeloma patients. Based on these results, we also planned to investigate the survival parameters (overall survival, OS) of patients with different breakpoints of the *NSD2* gene.

## 2. Materials and Methods

In our retrospective study, a total of 327 bone marrow (BM, *n* = 273), peripheral blood (PB, *n* = 34), or separated plasma cell (PC, *n* = 20) samples drawn at diagnosis, remission, and relapse were analyzed from 111 t(4;14) MM patients (54 males, 57 females; median age at diagnosis: 62 (27–83) years). Patients were consecutively diagnosed and treated at the Central Hospital of Southern Pest–National Institute of Hematology and Infectious Diseases (DPC-OHII, Budapest, Hungary) between January 2006 and December 2022. The international scoring system (ISS) was established at diagnosis [13]. Due to the retrospective nature of this study, data for 17p deletion or 1q amplification were not available in all cases; thus, the calculation of the revised international scoring system R-ISS or R2-ISS was not feasible for every case [16,17,18]. This study was conducted in accordance with the Declaration of Helsinki and was approved by the Institutional Review Board of DPC-OHII. Written informed consent was provided by all patients.

CD138-positive plasma cells were separated from bone marrow using EasySep™ Human Whole Blood and Bone Marrow CD138 Positive Selection Kit II (Stemcell^TM^ Technologies, Vancouver, BC, Canada).

Fluorescence in situ hybridization (FISH) testing was performed on bone marrow samples (*n* = 83, before 2018) or on separated CD138-positive plasma cells from bone marrow (*n* = 28, after 2018) using probes for translocation t(4;14), 17p deletion, and 1q amplification. Translocation t(4;14) was tested using Vysis IGH/FGFR3 Dual Fusion probes (Abbott Molecular, 01N69-020) and a Nikon Eclipse E400 epifluorescent microscope. For each sample, 200 bone marrow or plasma cells were scored, and the cutoff level was set at 10% for 17p deletion, 5% for 1q amplification, and 1–2% for translocation probes, according to the recommendation of the European Myeloma Network.

Flow cytometry analysis (FACS) was performed using a Becton Dickinson (BD) FACS Canto II cytometer (3-laser, 10-parameter configuration). The acquired data were analyzed using various software programs: FACS Diva v 6.0 (BD), Kaluza 2.2.1 (BeckmanCoulter, Brea, CA, USA), or Infinicyt 2.0 (Cytognos, Boston, MA, USA). The following markers were used: CD19 PC7 (Beckman (Brea, CA, USA), BCI-F-IM3628), CD20 APC-H7 (BD Biosciences (San Jose, CA, USA), 561172), CD27 PerCP-Cy5.5 (BD Biosciences, 560612), CD28, PerCP-Cy5.5 (BD Biosciences, 337181), CD38 APC CD38 (BD Biosciences, 345807), CD45 Krome Orange (Beckman, BCI-F-IM36294), CD56 FITC (BD Biosciences, 345811), CD81 APC-H7 (BD Biosciences, 656647), CD117 PE (BD Biosciences, 332785), CD138 VioBlue (Miltenyi Biotec (Bergisch-Gladbach, Germany), 130-119-843), Kappa FITC (BD Biosciences, 349516; intracellular staining), and Lambda PE (BD Biosciences, 349516; intracellular staining). Between 50,000 and 1,000,000 events (cells) were acquired in each test. Following an optimized gating strategy, we could exclude debris, specifically labeled cells, and duplets. In the results, we always enumerate the percentage of malignant plasma cells (of all living cells in the sample) and the ratio of these cells among all plasma cells.

RNA was freshly isolated from BM, PB, or PC samples using the TRIzol reagent (Invitrogen, Waltham, MA, USA, 15596018) without DNase treatment. RNA concentration was measured on a NanoDrop ND-1000 spectrophotometer. Three micrograms of isolated total RNA was employed for first-strand cDNA synthesis utilizing random hexa oligonucleotides with the High-Capacity cDNA Reverse Transcription Kit with RNase Inhibitor (Applied Biosystems, Waltham, MA, USA, 4374966) according to the manufacturer’s recommendations.

Initially, molecular diagnostic tests for breakpoint detection were performed using qualitative PCR with agarose gel electrophoresis. Our newly developed qPCR method operated on the following principles: the forward oligonucleotide and the hydrolysis probe target the JH region of the *IGH* gene, complemented in three separate reactions by three different, reverse oligonucleotides that align with the exons of the *NSD2* gene. At the time of diagnosis, three separate reactions were performed in parallel to identify the *NSD2* breakpoint specific to the patient. During MRD monitoring, the primer system specific to the previously identified breakpoint was exclusively used. Oligonucleotide design was performed using Primer3 [19] online and synthesized by ThermoFisher. Oligonucleotide sequences are summarized in Table 1. *ABL1* was used as a reference gene in parallel measurements, using PCR conditions similar to those of the *IGH::NSD2* amplification, with previously described oligonucleotides [20].

The qPCR was arranged on a LightCycler 480II instrument (Roche) using LightCycler 480 Probes Master Mix (Roche, 4707494001) in a final volume of 20 μL. Target (*IGH::NSD2*) and reference gene (*ABL1*) amplifications were performed in separate duplicate reactions with 240 ng of cDNA per well. The setup included a final concentration of 0.3 μM for both the forward (F) and reverse (R) primers, along with 0.2 μM for the probe. PCR parameters were as follows: cycling consisted of an initial denaturation at 95 °C for 10 min, followed by 50 cycles of 95 °C 15 s denaturation, and 63 °C 60 s annealing and extension. We checked the amplification sensitivity of *ABL1* using an ERM plasmid dilution series (Merck Life Sciences (Darmstadt, Germany), ERMAD623-6X0.6ML).

The system was later also optimized for droplet digital PCR (QX200™ AutoDG™ Droplet Digital™ PCR System, Bio-Rad, Hercules, CA, USA), utilizing ddPCR Supermix for Probes (Bio-Rad, 1863024) in a 22 μL final volume. In duplicates, 240 ng of cDNA was employed with oligonucleotides for both target and reference amplification in the same well. Oligonucleotide working concentrations were 0.9 μM for primers and 0.25 μM for hydrolysis probes. The hydrolysis probe for *ABL1* quantification was labeled with 5′HEX and 3′ ZEN/lowa Black FQ for dPCR. PCR parameters were as follows: initial denaturation at 95 °C for 10 min, followed by 39 cycles of 94 °C 30 s denaturation, and a 63 °C 60 s annealing and extension step. The acceptance criteria for dPCR measurements were the following: (1) total copy number > 10,000 for diagnostic or reference gene copy number > 32,000 for MRD samples to achieve at least 4.5-log sensitivity; (2) total droplet count > 15,000; (3) empty droplet count > 100. The dPCR was performed in 20–35 fusion gene-negative controls for each breakpoint to determine the limit of blank (LoB = mean of negative samples + 1.645× standard deviation (SD)) and the limit of detection (LoD = mean of negative samples + 3.3 × SD) [21].

Statistical analysis of the data was conducted using the SPSS software package (version 20.0; SPSS, Chicago, IL, USA). Comparisons of dichotomous variables were assessed using the Pearson χ² test or Fisher exact test, as appropriate; continuous variables were compared using Kruskal–Wallis or Mann–Whitney tests. The log-rank test was employed for the Kaplan–Meier method to compare overall survival. In multivariate survival analyses, Cox models were adjusted for age at diagnosis and ISS. We utilized the Bland–Altman test to compare the results between FACS and qPCR values, as well as to assess the concordance of the two quantitative systems (qPCR/dPCR) using GraphPad Prism 8. A significance level of *p* < 0.05 was considered indicative of a difference.

## 3. Results

### 3.1. Evaluation of the IGH::NSD2 qPCR System

To evaluate the clinical sensitivity of our newly developed qPCR system, we analyzed BM or PC samples obtained from 111 MM patients either at diagnosis or during progression, all of whom were confirmed as t(4;14)-positive by FISH. In addition, we compared our method with the qualitative PCR method previously described in the literature. We were able to identify the *IGH::NSD2* fusion transcript in 106 patients using qualitative PCR (95.5%). We detected 74 patients with the MB4-1 (66.5%), 11 patients with the MB4-2 (10%), and 21 patients with the MB4-3 (19%) breakpoint types, while in the remaining 5 cases (4.5%), we could not identify any single breakpoint type. With the exception of one patient, we were able to replicate these results using the qPCR system. We modified the forward primer and probe on the *IGH* gene while keeping the reverse primers unchanged. With this modified system, we managed to detect the *IGH::NSD2* fusion transcript in the patient who tested positive with qualitative PCR, while the other five patients continued to give negative results.

During qPCR, the slope and intercept of the calibration curve for the *IGH::NSD2* fusion mRNA expression level matched those of the calibration curve obtained from the same dilution series used for the reference gene; therefore, we calculated the fusion mRNA expression level using the delta Ct method. The sensitivity of the qPCR method was confirmed by a dilution series ranging from 1 to 100,000 times of a bone marrow sample collected at the time of diagnosis with close to 100% FISH t(4;14) positivity. The elevated sensitivity of our system became apparent in 31 samples where positivity could not be detected using qualitative PCR during the period when we performed qualitative and quantitative real-time PCR measurements simultaneously. However, we were able to demonstrate positivity using qPCR. We optimized the established qPCR system for dPCR technology as well. The measurements were conducted following the manufacturer’s standard specifications for the applied kit. Based on these, the method has an LOD (limit of detection) of <0.005% and an LOB (limit of blank) of <0.002% for dPCR. In comparison to qPCR, dPCR showed good concordance (R^2^ = 0.94, 95% limits of agreement: ^−^0.80–^+^0.63 in logarithmically transformed values). In the following calculations, the documented results are from the qPCR method, as it was performed in all of the samples, while dPCR was performed in a limited series (*n* = 34).

**Figure 1:**
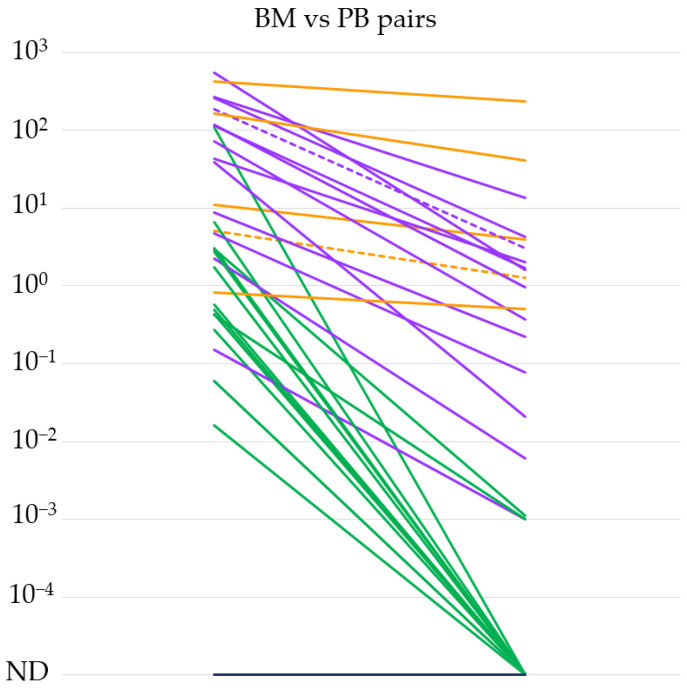
*IGH::NSD2* fusion transcript expression levels of bone marrow (BM) and peripheral blood (PB) sample pairs (*n* = 34). The orange lines indicate differences of less than 1, the purple lines between 1 and 3, and the green lines of more than 3 orders of magnitude. Extramedullary MM cases are marked with dashed lines. ND = not detectable.

Based on the high sensitivity of our qPCR method, we tested the *IGH::NSD2* fusion transcript expression level in 34 BM and PB sample pairs taken at the same time point from 25 different patients. We summarize our results in Figure 1. In the case of 20 BM-PB sample pairs, the *IGH::NSD2* fusion transcript expression was measurable even from the PB samples. In the remaining 14 pairs, only the BM samples were positive. In general, we could conclude that the *IGH::NSD2* fusion transcript in the PB samples (median 0.0035%, range: 0–235) was measurable at a 3-log lower sensitivity compared to the BM samples (median 3.9%, range: 0–543). Interestingly, in one out of five cases where a difference of less than 1 log was detectable between the BM and PB pairs, extramedullary MM was demonstrated. Furthermore, extramedullary involvement was observed in one more case where we detected a difference ranging between one and three orders of magnitude between BM and PB.

### 3.2. Survival Data of Patients According to the Different NSD2 Breakpoint Types

Having the opportunity to process clinical and treatment data from 104 patients, we analyzed overall survival parameters according to the *NSD2* breakpoint types. The median follow-up for the entire cohort was 60 months. Patient characteristics are summarized in Table 2. There was no significance observed in any of the examined clinical parameters between the groups of patients with different breakpoints.

**Figure 2:**
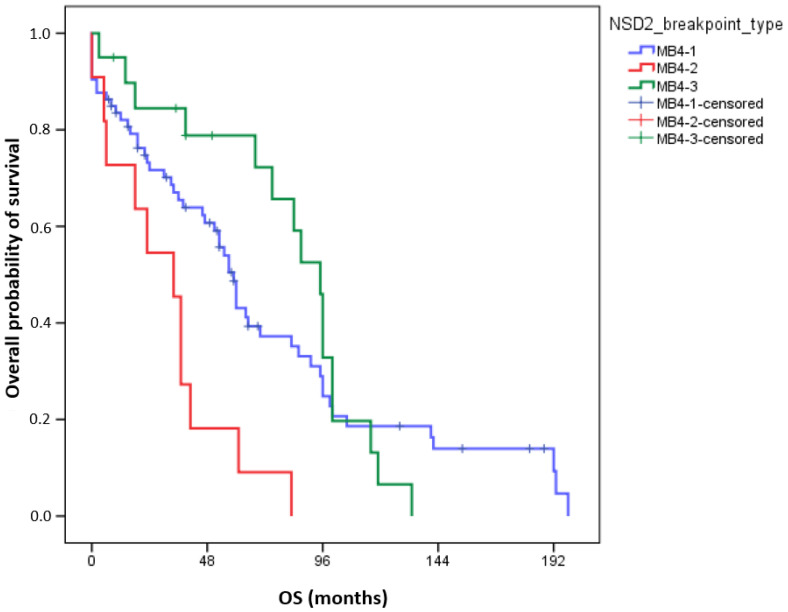
Overall survival (OS) of 104 MM patients according to different *IGH::NSD2* breakpoint types. The number of events for groups MB4-1, MB4-2, and MB4-3 is 54, 11, and 16, respectively.

Interestingly, in univariate analyses, adverse OS was observed in the MB4-2 breakpoint group (72-month OS MB4-1: 37.2 ± 6.3%; MB4-2: 9.1 ± 8.7%; MB4-3: 72.2 ± 10.7%, *p* = 0.003, Figure 2). In pairwise comparisons, the OS of MB4-2 significantly differed from MB4-1 (*p* = 0.005) and from MB4-3 (*p* < 0.001), but the OS of MB4-1 and MB4-3 were not different (*p* = 0.6). Using multivariate analyses, the presence of the MB4-2 breakpoint proved to be an independent adverse factor influencing OS aside from age and ISS (*p* = 0.018). Uni- and multivariate Cox regression results are summarized in Table 3.

## 4. Discussion

According to our knowledge, there is no proven sensitive and quantitative method for *IGH::NSD2* expression measurements, even though this fusion mRNA represents a founder driver in MM in 10–15% of all MM patients. The t(4;14) translocation leads to elevated *NSD2* expression and uncontrolled cell proliferation. A qPCR method was previously employed with one reaction for each breakpoint type, though there are no available data on its sensitivity [14]. Therefore, we developed a highly sensitive qPCR system capable of detecting these *NSD2* breakpoints. During the initial patient assessment, we were able to identify the specific breakpoint in three separate reactions. Subsequently, during MRD measurements, a single assay was sufficient for determining the expression of both the target and housekeeping genes. Our patient group consisted of 111 patients, out of which 106 (95%) were positive for the *IGH::NSD2* fusion gene with molecular genetic methods. In five patients, despite the t(4;14) translocation detected by FISH analysis, we were unable to identify *IGH::NSD2* fusion mRNA using either the basic or quantitative PCR methods. By redesigning the forward primer and TaqMan probe (with a 70 and 25 basepair shift) targeting the *IGH* gene, we were able to successfully detect fusion mRNA using qPCR in a patient where the qualitative PCR indicated a positive translocation, so we could achieve 100% concordance between our method and the qualitative PCR system published before. The clinical sensitivity of both molecular methods was 95%.

A recently published study reported detailed data from more than one hundred MM patients. They confirmed t(4;14) positivity using FISH and WGS techniques, but fusion transcripts could only be identified in 87.3% of cases [18]. These findings align with the detection rate observed within the patient cohort we examined and may suggest the presence of a rare IGH gene rearrangement or, more likely, deletions within the *IGH* or *NSD2* genes. In their cohort, an internal deletion downstream of the translocation breakpoint resulted in the loss of exon 2 in 9 out of 109 patients. However, this cannot solely account for the absence of positivity observed, even with the reverse primers targeting the third and fourth exons of the *NSD2* gene in our system.

Although we demonstrated a good concordance between the two quantitative methods, suggesting that qPCR and dPCR could be used interchangeably, we assessed the sensitivity of the developed qPCR method through various approaches. The qPCR analytical sensitivity proved reliable within the 1:100,000 range. We demonstrated this by diluting mRNA isolated from nearly 100% t(4;14)-positive BM samples using FISH analysis. Furthermore, we optimized our system for the droplet-digital platform, enabling reliable and comparable results during patient follow-up across different laboratories. In our investigation of 36 peripheral blood samples from 25 patients, this sensitive system demonstrated the presence of affected plasma cells in the circulation in several cases, particularly in instances of extramedullary involvement. In general, we conclude that the *IGH::NSD2* fusion transcript in peripheral blood samples can be measured with a sensitivity 1000 times lower than that in BM samples. This more convenient and less invasive PB sampling for measurable residual disease monitoring could potentially replace the need for frequent BM sample collection. High-sensitivity measurable residual disease evaluation can be used to drive treatment-free remission periods for afflicted patients, especially if MRD negativity is sustained. As in t(4;14) myeloma, the neurotoxic bortezomib maintenance therapy is widely used, and treatment-free periods are precious for patients with low-grade ongoing polyneuropathy.

The mechanism by which clonal plasma cells causing myeloma exit the bone marrow is not entirely understood. Several publications suggest that subclones that are detectable extramedullarily may have more complex cytogenetic and molecular genetic backgrounds than the affected plasma cells present in the bone marrow [22]. Based on this, the examination of *IGH::NSD2* fusion transcripts in peripheral blood samples cannot definitively determine whether the signal originates from circulating tumor cells derived from the bone marrow or if it indicates the presence of extramedullary manifestations. Among the BM-PB pairs indicated in Figure 1, extramedullary involvement was proven in two cases at the time of sampling. In one case, the BM vs. PB fusion mRNA level difference did not reach an order of magnitude difference, while in the other case, the difference in *IGH::NSD2* expression was within the range of 1–3 orders of magnitude. Based on these, it can be presumed that the measurable *IGH::NSD2* fusion mRNA in peripheral blood originates from the original abnormal plasma cells that exited the bone marrow. However, this could only be confirmed through more detailed FISH and multigene NGS examinations.

The distribution of breakpoints in our cohort exhibited a strong correlation with data reported by other groups. However, Li et al. presented differing results, possibly attributed to their considerably small sample size (Table 4).

Several attempts have been made to correlate survival differences based on various breakpoints observed in the *IGH::NSD2* fusion gene. Most studies grouped the MB4-2 and MB4-3 breakpoint types together, which is sensible because in the presence of the MB4-1 breakpoint, a full-length *NSD2* transcript can be generated. Keats et al. did not identify any significant differences associated with breakpoint types. Li et al., in a similarly small patient group, reported that patients with the MB4-1 breakpoint type had a better overall survival outcome compared to the combined MB4-2 and MB4-3 groups, likely attributed to a more favorable response to bortezomib. However, they did not find any association with progression-free survival. In a recently published paper, Stong et al. also revealed that patients with MB4-1 breakpoints expressing full-length protein showed significantly better overall survival rates compared to those expressing truncated protein.

Lazareth et al., in a larger cohort of 256 patients, discovered that those with the MB4-2 breakpoint responded better to initial treatment but later developed chemo-resistant relapses and experienced poorer outcomes. In our cohort of 104 individuals with adequate clinical information for overall survival calculations, we initially grouped the two patient groups with truncated protein as well, but, similarly, we found no significant difference compared to those expressing full-length protein in terms of overall survival. However, upon stratifying the patients based on the three breakpoints, our findings echoed those of Lazareth: individuals expressing MB4-2 exhibited significantly adverse survival within our studied patient cohort.

Our article naturally exhibits certain weaknesses. Owing to the prolonged patient recruitment period, clinical- and treatment-related data for early-stage patients are only partially available. Therefore, we did not have the opportunity to calculate R-ISS or R2-ISS. We were only able to calculate the ISS classification for 87 patients. Moreover, additional chromosomal abnormalities, notably 1q amplification and 17p deletion, recognized for their negative impact on patients’ survival, were only accessible within a subset of the studied patient group. We have been able to systematically examine BM and PB pairs using the new qPCR test only in the last four years. In previous years, bone marrow samples were either not available or arrived irregularly during patient follow-ups. Therefore, retrospectively, we did not have the opportunity for detailed MRD measurements. Due to these reasons, we were unable to perform progression-free survival calculations. Nevertheless, despite all this, we believe that OS values alone are valuable and comparable to other research groups’ results, as these groups also regularly report OS values alongside progression-free survival. Clinical sensitivity did not reach 100%, but the evaluation of t(4;14) positivity is routinely performed using the FISH method at the time of diagnosis.

## 5. Conclusions

Our new method provides an opportunity for the execution of a simple and sensitive minimal residual disease detection process in a significant proportion of multiple myeloma patients who have the translocation t(4;14). This can be achieved even using peripheral blood samples in any molecular genetic laboratory. Its utilization can facilitate regular MRD monitoring during patient follow-up.

The literature lacks consensus regarding the survival outcomes of patients with various *NSD2* breakpoints. Our data align with findings suggesting that patients with the MB4-2 breakpoint type exhibit adverse overall survival.

## Figures and Tables

**Table 1:** Summary of the applied oligonucleotides for qPCR/dPCR system.

**Oligonucleotides for *IGH* (reference sequence: NG_001019.6)**	
Forward primer for JH region	5′-GCCCTTGTTAATGGACTTGGA-3′
Hydrolysis probe for JH region	5′-FAM-TCCATGCCAAAGCTTTGCAAGGC-TAMRA-3′ (for qPCR)
	5′-FAM-TCCATGCCAAAGCTTTGCAAGGC-ZEN/lowa Black FQ-3′ (for dPCR)
**Oligonucleotides for *NSD2* (reference sequence: NG_009269.1)**	
Reverse primer for exon 2	5′-GGATTTCTGGTGCCTGCTTC-3′
Reverse primer for exon 3	5′-CCACACCAAATCACCAACGT-3′
Reverse primer for exon 4	5′-CTCCTTCAAAAGCTACGAGGC-3′

**Table 2:** Summary of the important clinical parameters of the examined patients according to the different *NSD2* breakpoint types.

	Overall	MB4-1	MB4-2	MB4-3	*p*
N (%)	104	73 (70.5)	11 (10.5)	20 (19)	
Sex F/M	53/51	38/35	5/6	10/10	0.916
Age at diagnosis (year) median (interquartile range)	62 (17)	62 (17)	64 (14)	60 (15)	0.445
Heavy-chain isotype IgG IgA no heavy chain	104 50 50 4	37 33 3	3 8 0	10 9 1	0.53
Light chain kappa lambda no data	101 64 37 3	43 27 3	6 5 0	15 5 0	0.557
LDH (U/L) median (interquartile range)	221 (330)	220 (338)	266 (371)	210 (270)	0.838
Albumin (g/L) median (interquartile range)	32 (38)	29 (37)	34 (44)	34 (36)	0.191
Hemoglobin (g/L) median (interquartile range)	86 (107)	85 (106)	91 (118)	88 (87)	0.737
beta-2 microglobulin (μg/mL) median (interquartile range) from 85 patients	4.51 (5.14)	4.35 (4.98)	5.25 (13.35)	3.87 (6.47)	0.764
ISS	87	60	9	18	0.73
I	32 (37.6%)	22 (36.6%)	3 (33.4%)	7 (38.9%)	
II	20 (23.5%)	12 (20%)	2 (22.2%)	6 (33.3%)	
III	35 (41.2%)	26 (43.4%)	4 (44.4%)	5 (27.8%)	
Additional cytogenetics (1q amplification/17p deletion)	64	42	6	12	0.722
Patients treated with autologous stem cell transplantation	49/79	32/57	3/5	14/17	0.147

**Table 3:** Cox proportional hazard model for overall survival according to the different *NSD2* breakpoint types.

	Univariate Analyses (*n* = 104)		Multivariate Analyses (*n* = 87)	
	Hazard ratio (95% CI)	*p*	Hazard ratio (95% CI)	*p*
Age at diagnosis (in years)	1.033 (1.011–1.055)	0.003	1.024 (0.999–1.049)	0.061
ISS (ISS I as reference)	1.503 (1.128–2.003)	0.005	1.518 (1.139–2.024)	0.004
Breakpoint types (MB4-1 as reference)		0.006		0.018
MB4-1 vs. MB4-2	2.839 (1.442–5.59)	0.003	2.897 (1.345–6.237)	0.007
MB4-1 vs. MB4-3	0.855 (0.484–1.509)	0.588	0.906 (0.488–1.682)	0.754

**Table 4:** Comparison of number of patients with different *NSD2* breakpoint types from different investigations. (* indicates a significant difference in comparison to our cohort).

Breakpoint Type	Our Cohort *n* (%)	Keats *n* (%) [23]	Lazareth *n* (%) [11]	Li *n* (%) [12]	Stong *n* (%) [24]
MB4-1	74 (69.8%)	32 (71.1)	159 (62.1)	25 (47.2)	66 (60.5)
MB4-2	11 (10.4%)	6 (13.3)	54 (21.1)	12 (22.6)	16 (14.7)
MB4-3	21 (19.8%)	7 (15.6)	43 (16.8)	16 (30.2)	27 (24.8)
Total	106	45	256	53	109
*p*=		0.7552	0.0531	0.016 *	0.3514

## Data Availability

The data presented in this study are available on request from the corresponding author.
